# Impact of postnatal dexamethasone timing on preterm mortality and bronchopulmonary dysplasia: a propensity score analysis

**DOI:** 10.1183/13993003.00825-2023

**Published:** 2023-10-19

**Authors:** T’ng Chang Kwok, Lisa Szatkowski, Don Sharkey

**Affiliations:** Centre for Perinatal Research, Population and Lifespan Science, School of Medicine, University of Nottingham, Queen's Medical Centre, Nottingham, UK

## Abstract

**Background:**

Postnatal dexamethasone (PND) is used in high-risk preterm infants after the first week of life to facilitate extubation and prevent bronchopulmonary dysplasia (BPD) but the optimal treatment timing remains unclear. Our objective was to explore the association between the timing of PND commencement and mortality and respiratory outcomes.

**Methods:**

This was a retrospective National Neonatal Research Database study of 84 440 premature infants born <32 weeks gestational age from 2010 to 2020 in England and Wales. Propensity score weighting analysis was used to explore the impact of PND commenced at three time-points (2–3 weeks (PND^2/3^), 4–5 weeks (PND^4/5^) and after 5 weeks (PND^6+^) chronological age) on the primary composite outcome of death before neonatal discharge and/or severe BPD (defined as respiratory pressure support at 36 weeks) alongside other secondary respiratory outcomes.

**Results:**

3469 infants received PND. Compared with PND^2/3^, infants receiving PND^6+^ were more likely to die and/or develop severe BPD (OR 1.68, 95% CI 1.28–2.21), extubate at later postmenstrual age (mean difference 3.1 weeks, 95% CI 2.9–3.4 weeks), potentially require respiratory support at discharge (OR 1.34, 95% CI 1.06–1.70) but had lower mortality before discharge (OR 0.38, 95% CI 0.29–0.51). PND^4/5^ was not associated with severe BPD or discharge respiratory support.

**Conclusions:**

PND treatment after 5 weeks of age was associated with worse respiratory outcomes although residual bias cannot be excluded. A definitive clinical trial to determine the optimal PND treatment window, based on early objective measures to identify high-risk infants, is needed.

## Tweetable abstract

**The ideal timing of postnatal dexamethasone to prevent BPD is unclear. After minimising residual confounding, first dexamethasone use at >5 weeks old is associated with a higher incidence of severe BPD/death and later extubation than use at 2–3 weeks old.** https://bit.ly/3s6UtOT

## Introduction

Preterm infants born <32 weeks gestational age (GA) are at high risk of developing bronchopulmonary dysplasia (BPD) which is associated with a higher risk of mortality and morbidity [1], chronic pulmonary diseases in adulthood [2, 3], as well as long-term neurodevelopmental impairment [2]. With increasing BPD severity, the risk of late death after 36 weeks postmenstrual age (PMA), severe respiratory morbidity or moderate/severe neurodevelopmental impairment is up to seven times higher compared with preterm infants without BPD [4].

Postnatal dexamethasone (PND) is frequently used [5] to facilitate the extubation of preterm infants and reduce BPD and mortality risk [6, 7]. However, there is insufficient evidence as to which group of infants would benefit from PND and the optimal time to start treatment [6, 7], leading to variation in practice [8]. PND use, especially in the first week of life, in infants at low risk of BPD is associated with poor neurodevelopmental outcomes and cerebral palsy [9], alongside other adverse effects including hypertension and gastrointestinal perforation [10]. Conversely, delaying treatment may expose infants likely to benefit from treatment to a longer duration of invasive ventilation leading to further lung inflammation and injury [11], so missing the therapeutic window.

Most studies to date examining the optimal timing of PND treatment have been small, underpowered or terminated early [6, 7, 12]. A recent Cochrane review found that PND commenced after 7 days of life in preterm infants reduced mortality and BPD risk (553 infants, 12 trials) but was unable to examine optimal timing [7]. Additionally, a network meta-analysis of 14 corticosteroid BPD prevention regimes suggested a moderate dose of PND on days 8–14 of life was most effective (660 infants) in preventing mortality or BPD [12]. Conversely, another network meta-analysis of five corticosteroids for BPD (6747 infants) found aggressive early PND initiation in the first week of life to be beneficial, but did not break down the late (after 7 days) PND group to explore optimal timing [13]. However, in all three analyses combined, only 139 infants were recruited since 2003, with most studies undertaken 20–35 years ago, before modern neonatal intensive care approaches to reduce mortality and BPD. Therefore, this study aims to explore the impact of the chronological age when PND was commenced on preterm mortality and respiratory outcomes in a large contemporary national cohort.

## Material and methods

### Study design

This population-based retrospective cohort study uses de-identified data from the National Neonatal Research Database (NNRD), a population-level dataset containing detailed information entered at the point of care. The data cover the entire clinical stay across multiple neonatal units, encompassing once-only demographic information including gestation, birthweight and sex as well as daily data including respiratory support and drugs received (without doses). Over 90% of neonatal units in England in 2010 contributed data to the NNRD, with 100% coverage in England and Wales by 2012 and 2014, respectively. Ethical approval was granted by the Sheffield Research Ethics Committee (19/YH/0115). The study was reported using the Strengthening the Reporting of Observational Studies in Epidemiology guidelines [14], Lederer *et al.* [15] and propensity score checklists [16].

### Patient population

We included all infants born <32 weeks GA admitted to 185 neonatal units in England and Wales from 1 January 2010 to 31 December 2020. In line with current PND recommendations in premature infants [10], infants who received PND after the first 7 days of life but before 36 weeks PMA and who were invasively ventilated when PND was first commenced were included in the propensity score analysis. Infants with a birthweight for GA z-score >4sd above or below the mean, or who were discharged to nonparticipating units, were excluded as they likely represent erroneous or incomplete entries, respectively. Infants with major congenital anomalies as defined previously (supplementary table S1) [17] or missing data on our primary composite outcome of death before discharge and/or severe BPD were also excluded.

### Definition of clinical practices and outcomes

PND use was defined as >2 consecutive days of receiving PND, based on a recent survey [8], to ensure its intended use to treat or prevent BPD. A 7-day washout period was used to define new PND courses. The primary outcome was the composite outcome of death before discharge and/or severe BPD. Secondary respiratory outcomes included the individual outcomes of death, BPD, severe BPD, respiratory support requirement at discharge, duration of invasive ventilation, percentage of infants who were successfully extubated for at least 7 days within 14 days of receiving PND and PMA when successfully extubated.

Severe BPD was defined using the grade 2/3 BPD definition by Jensen *et al.* [4], which is respiratory pressure support (noninvasive (including high flow >2 L·min^−1^) and invasive ventilation) requirement at 36 weeks PMA due to its stronger association with long-term pulmonary and neurodevelopmental sequelae [4, 18]. These were assessed over 3 days (36 weeks PMA±1 day) to allow for missing data or fluctuating requirements. If infants were discharged before 36 weeks PMA, respiratory support at discharge was used. Infants who died before discharge were excluded from the respiratory support at discharge and BPD definitions. Further definitions of the variables are described in supplementary table S2.

### Statistical analyses

All statistical analyses were performed using Stata SE version 17 [19] and RStudio [20] with Bonferroni-corrected significance level for multiple testing. Summary statistics, such as median, interquartile range (IQR) and percentage, were used to describe the infant characteristics in the overall cohort and those who received PND. Trends over time were analysed using the Chi-squared test for trend [21] and a Wilcoxon rank-sum test extension [22] for categorical and continuous data, respectively.

**FIGURE 1:**
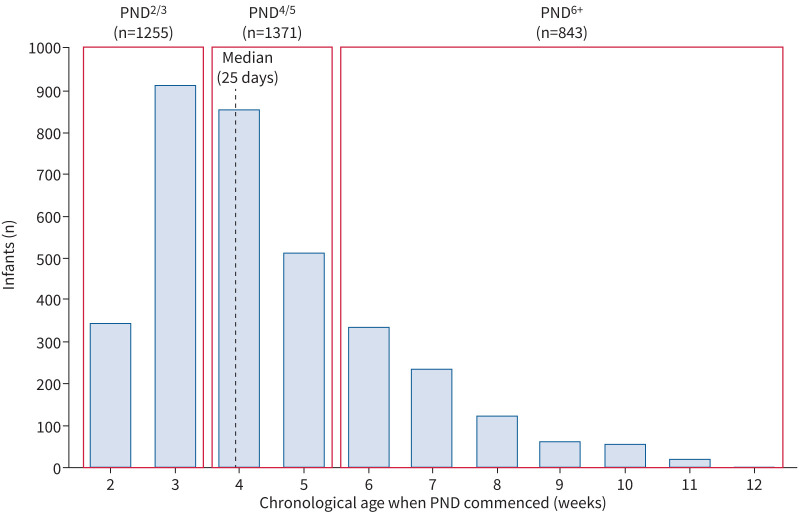
Histogram showing the chronological age when postnatal dexamethasone (PND) was commenced in 3469 infants who met the study inclusion criteria with a median of 25 days and the three groups of infants receiving PND at 2–3 weeks (PND^2/3^), 4–5 weeks (PND^4/5^) and after 5 weeks (PND^6+^) chronological age. PND was commenced beyond 12 weeks in two infants (0.06%).

Propensity score weighting analysis was performed using the twang package [23, 24] to minimise bias by confounding in exploring the association of the timing of commencing PND on respiratory outcomes. The cohort was split into three groups based on the chronological age they received PND: 2–3 weeks (PND^2/3^), 4–5 weeks (PND^4/5^) and after 5 weeks (PND^6+^). The three groups were chosen to assess the impact of commencing PND before, at and after the median chronological age when PND was commenced (figure 1).

The propensity for the infants to be assigned to each of the three PND groups was then estimated based on *a priori* variables (supplementary table S2) [10, 25] using the generalised boosted model, a machine learning approach which relies on iterative tree-based regression models [24]. The estimated propensity scores were subsequently used as inverse weights in estimating the treatment effects based on the inverse probability of treatment weighting (IPTW) approach [16]. The success of the IPTW approach was assessed by examining the balance of the *a priori* variables across the three groups. Missing values (supplementary table S3) were controlled for by including missing value indicators and balancing rates of missingness in the three groups. Finally, the difference in weighted means was used to estimate the association between treatment groups and outcomes as the *a priori* variables were balanced across the three groups after IPTW. The propensity score weighting approach is described in further detail in the supplementary material.

Four sensitivity analyses were performed. 1) To minimise potential lead time bias, infants who received PND after 32 weeks PMA were excluded to allow PND at least 4 weeks to take effect before BPD was diagnosed at 36 weeks PMA. 2) To minimise survival bias, infants who died before 6 weeks of age were excluded so that infants will have at least survived to the earliest time-point as infants in the late PND^6+^ group. As the GA at birth was not balanced after IPTW for the first two sensitivity analyses, a double adjustment [26] was performed for GA at birth. 3) To minimise unmeasured bias [27], infants with propensity scores outside the 5–95th centile ranges of all three PND groups were excluded. 4) To partially account for PND courses for BPD that may have been terminated early, PND use was defined as at least 7 consecutive days of treatment, which is two-thirds of the duration of the most commonly used PND regime for BPD [8].

## Results

### Infant cohort

**FIGURE 2:**
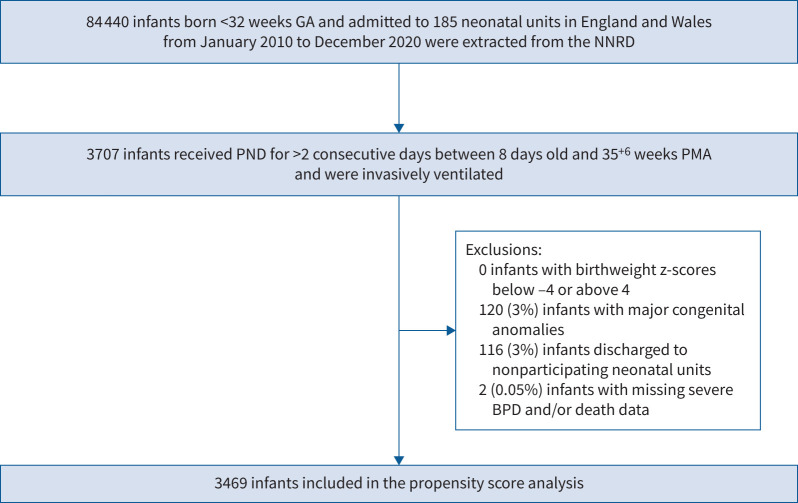
Participant flow diagram for the analysis of the trend of postnatal dexamethasone (PND) use from 2010 to 2020 as well as the propensity score analysis on the timing of commencing PND. GA: gestational age; NNRD: National Neonatal Research Database; PMA: postmenstrual age; BPD: bronchopulmonary dysplasia.

84 440 premature infants born <32 weeks GA were admitted into 185 neonatal units within the NNRD from 2010 to 2020. 3469 (4%) infants fulfilled the inclusion criteria for the propensity score analysis of PND use (figure 2 and table 1). 541 (16%) of these infants died at a median (IQR) chronological age of 49 (28–91) days. 2827 (81%) infants developed the composite outcome of death and/or severe BPD. Among the 2928 survivors to discharge, 2018 (69%) infants required respiratory support at discharge (table 1).

**TABLE 1:** Demographics and clinical characteristics of all infants extracted from the National Neonatal Research Database as well as infants who received a course of postnatal dexamethasone (PND)

	**All infants (n=84 440)**	**Received PND^#^ (n=3469)**
**Gestation at birth (weeks)** ^¶^	29^+3^ (27^+2^–30^+6^)	25^+0^ (24^+1^–26^+2^)
**Birthweight (g)** ^¶^	1197 (900–1490)	700 (609–815)
**Small for GA** ^¶,+^	12 589 (15)	766 (22)
**Male^¶^**	46 118 (55)	2077 (60)
**Received ANCs^¶^**	75 637 (90)	3125 (91)
**Received surfactant**	52 225 (62)	3360 (97)
**Duration of invasive ventilation (days)**	2 (0–6)	38 (27–52)
**Death^§^**	7529 (9)	541 (16)
**Severe BPD or death^¶,§^**	19 399 (23)	2827 (81)
**Respiratory support at 36 weeks PMA^¶,##^**		
None	52 453 (68)	133 (5)
Oxygen only	12 157 (16)	509 (17)
Pressure support	11 870 (15)	2286 (78)
**Respiratory support at discharge^¶,##^**		
None	64 077 (83)	893 (31)
Oxygen only	10 051 (13)	1637 (56)
Pressure support	2048 (3)	381 (13)

Data are presented as median (interquartile range) or n (%). GA: gestational age; ANC: antenatal corticosteroid; BPD: bronchopulmonary dysplasia; PMA: postmenstrual age. ^#^: PND was defined as dexamethasone use for >2 consecutive days in ventilated infants between 8 days chronological age and 36 weeks PMA; ^¶^: infants with missing data in the full cohort for the following variables: gestation n=2 (<0.01%), birthweight n=11 (0.01%), small for GA n=116 (0.1%), sex n=62 (0.1%), ANCs n=715 (0.9%), severe BPD and/or death n=431 (0.5%), respiratory support at 36 weeks PMA n=431 (0.6%) and respiratory support at discharge n=735 (1.0%); ^+^: small for GA was defined as birthweight <10th centile based on the UK–World Health Organization growth chart [28]; ^§^: death was defined as death before discharge from the neonatal unit; ^##^: infants who died before discharge were excluded from the analysis of respiratory support at 36 weeks PMA and discharge, respectively.

### Dexamethasone use

PND use in ventilated infants between 8 days of age and 36 weeks PMA increased from 3% in 2010 to 5% in 2020 (p<0.001) and was commenced earlier from a median (IQR) of 28 (20–40) days chronological age in 2010 to 24 (18–32) days in 2020 (p<0.001). There was an increasing trend in the percentage of infants receiving multiple PND courses from 25% in 2010 to 34% in 2020 (p=0.04). Infants born at earlier gestations (p<0.001) and lower birthweights (p<0.001) were more likely to receive PND later (table 2 and supplementary table S4).

**TABLE 2:** Demographics and clinical characteristics of the 3469 infants who received postnatal dexamethasone (PND) stratified by the chronological age they first received PND

**Age when PND^#^ started (weeks)**	**GA at birth (weeks)**	**Birthweight (g)**	**Male**	**ANCs** ^¶^	**Surfactant**	**Duration of first PND course (days)**	**Total time on PND (days)**	**Multiple PND courses**
**2 (n=344)**	25^+1^ (24^+1^–26^+4^)	730 (630–855)	212 (62)	303 (88)	338 (98)	10 (6–19)	14 (8–29.5)	115 (33)
**3 (n=911)**	25^+1^ (24^+1^–26^+2^)	705 (600–820)	552 (61)	802 (88)	878 (96)	11 (8–17)	15 (10–27)	323 (35)
**4 (n=854)**	25^+2^ (24^+2^–26^+3^)	710 (614–830)	517 (61)	794 (93)	840 (98)	10 (8–15)	13 (9–23)	250 (29)
**5 (n=517)**	25^+0^ (24^+1^–26^+1^)	705 (617–810)	306 (59)	471 (92)	498 (96)	10 (7–14)	12 (9–20)	121 (23)
**6 (n=337)**	25^+0^ (24^+1^–26^+1^)	685 (600–780)	200 (59)	307 (91)	317 (94)	10 (7–13)	12 (9–19)	81 (24)
**7 (n=237)**	24^+5^ (24^+0–^26^+1^)	665 (580–780)	141 (59)	206 (88)	231 (97)	10 (7–13)	12 (9–21)	63 (27)
**8 (n=121)**	25^+0^ (24^+1^–26^+2^)	680 (592–815)	66 (55)	107 (90)	116 (96)	10 (7–14)	12 (9–19)	29 (24)
**9 (n=67)**	24^+5^ (24^+0^–25^+4^)	658 (600–770)	37 (55)	59 (88)	66 (99)	11 (7–12)	11 (9–16)	16 (24)
**≥10 (n=81)**	24^+3^ (23^+6^–25^+0^)	650 (570–750)	46 (57)	76 (95)	76 (94)	10 (6–14)	12 (7–23)	21 (26)
**p-value for trend^+^**	<0.001*	<0.001*	0.1	0.2	0.06	0.001*	<0.001*	<0.001*

Data are presented as median (interquartile range) or n (%), unless otherwise stated. GA: gestational age; ANC: antenatal corticosteroid. ^#^: a course of PND was defined as PND use for >2 consecutive days in ventilated infants between 8 days chronological age and 36 weeks postmenstrual age; ^¶^: data on complete ANC course were missing for 17 (0.5%) infants; ^+^: extension of Wilcoxon rank-sum test or Chi-squared test for trend was performed to examine the association of continuous or categorical variables, respectively, by the chronological age of infants first receiving PND in weeks. *: statistically significant after Bonferroni correction.

### Propensity score analysis

All *a priori* variables were balanced across the three groups after weighting (supplementary table S4, supplementary figures S1 and S2, and supplementary material). After weighting, PND^6+^ infants were more likely to develop the composite outcome of death before discharge and/or severe BPD (OR 1.68, 95% CI 1.28–2.21) but were less likely to die before discharge (OR 0.38, 95% CI 0.29–0.51) when compared with PND^2/3^ infants. In infants who survived to discharge, PND^6+^ infants received a longer duration of invasive ventilation (mean difference 17.5 days, 95% CI 15.3–19.7 days) and were extubated at a later PMA after commencing PND (mean difference 3.1 weeks, 95% CI 2.9–3.4 weeks) when compared with PND^2/3^ infants. Although not achieving the conservative Bonferroni-corrected statistical significance, there was an increasing trend of respiratory support requirement at neonatal discharge in PND^6+^ infants who survived to discharge than in PND^2/3^ infants (OR 1.34, 95% CI 1.06–1.70). There was no statistically significant difference in the odds of BPD among the three PND groups. Compared with PND^2/3^ (reference), PND^4/5^ and PND^6+^ infants were more likely to be successfully extubated within 14 days of starting PND, but this did not translate into differences with severe BPD or respiratory support at discharge (table 3).

**TABLE 3:** Neonatal outcomes between infants who received postnatal dexamethasone (PND) at the chronological age of 2–3 weeks (PND^2/3^), 4–5 weeks (PND^4/5^) and after 5 weeks (PND^6+^) in the unweighted (n=3469) and weighted infant cohort (effective sample size (ES)=3032)

	**Unweighted cohort**			**Weighted cohort^#^**				
	**PND^2/3^** **(n=1255)**	**PND^4/5^** **(n=1371)**	**PND^6+^** **(n=843)**	**PND^2/3^** **(ES=1098)**	**PND^4/5^** **(ES=1298)**	**PND^6+^** **(ES=636)**	**Treatment effect** **OR/MD (95% CI)**	**p-value**
**Primary outcome**								
Severe BPD and/or death	991 (79.0)	1098 (80.1)	738 (87.5)	79.6	79.9	86.8	PND^2/3^: reference PND^4/5^: 1.02 (0.84–1.24) PND^6+^: 1.68 (1.28–2.21)	0.851 <0.001*
**Secondary outcomes**								
Death	273 (21.8)	177 (12.9)	91 (10.8)	21.6	13.1	9.6	PND^2/3^: reference PND^4/5^: 0.55 (0.44–0.68) PND^6+^: 0.38 (0.29–0.51)	<0.001* <0.001*
Severe BPD^¶^	718 (73.1)	921 (77.1)	647 (86.0)	74.0	76.9	85.4	PND^2/3^: reference PND^4/5^: 1.17 (0.95–1.43) PND^6+^: 2.05 (1.55–2.71)	0.135 <0.001*
BPD^¶^	928 (94.5)	1138 (95.3)	729 (96.9)	94.8	95.3	96.1	PND^2/3^: reference PND^4/5^: 1.11 (0.74–1.66) PND^6+^: 1.34 (0.77–2.34)	0.608 0.304
Duration of invasive ventilation (days)^¶^	30 (22–43)	35 (28–45)	51 (41–64)	30 (22–44)	35 (28–46)	49 (40–60)	PND^2/3^: reference PND^4/5^: 4.3 (2.6–6.0) PND^6+^: 17.5 (15.3–19.7)	<0.001* <0.001*
Successful extubation within 14 days of starting PND^¶^	609 (62.0)	899 (75.3)	591 (78.6)	60.2	74.7	79.6	PND^2/3^: reference PND^4/5^: 2.0 (1.6–2.4) PND^6+^: 2.6 (2.0–3.3)	<0.001* <0.001*
PMA when successfully extubated after receiving PND (weeks)^¶^	29^+5^ (28^+4^–31^+2^)	30^+4^ (29^+3^–31^+6^)	32^+6^ (31^+3^–34^+3^)	29^+5^ (28^+4^–31^+3^)	30^+4^ (29^+2^–31^+6^)	32^+6^ (31^+3^–34^+3^)	PND^2/3^: reference PND^4/5^: 0.7 (0.5–0.9) PND^6+^: 3.1 (2.9–3.4)	<0.001* <0.001*
Respiratory support at discharge^¶^	658 (67.0)	807 (67.6)	553 (73.5)	67.4	67.8	73.2	PND^2/3^: reference PND^4/5^: 1.04 (0.86–1.25) PND^6+^: 1.34 (1.06–1.70)	0.713 0.015

Data are presented as median (interquartile range) or n (%), unless otherwise stated. BPD: bronchopulmonary dysplasia; OR: odds ratio; MD: mean difference. ^#^: only the percentage of infants with the respective categorical neonatal outcomes was depicted for the weighted infant cohort; ^¶^: infants who died before discharge were excluded from the analysis. *: statistically significant after Bonferroni correction.

### Sensitivity analyses

All four sensitivity analyses found similar findings of more severe BPD and extubation at a later PMA in PND^6+^ infants than in PND^2/3^ infants (supplementary tables S5–S8). In the first two sensitivity analyses, more PND^6+^ infants required respiratory support at discharge than in PND^2/3^ infants, achieving Bonferroni-corrected statistical significance (supplementary tables S5 and S6). The odds of death among all three groups were not different when excluding infants who died before 6 weeks old to minimise survival bias (supplementary table S6).

## Discussion

This large population-based cohort study, representing >90% of live births of premature infants born <32 weeks GA in England and Wales, describes PND use and the association between the timing of PND commencement and respiratory morbidities and mortality. This provides a true reflection of current practices and valuable data for healthcare professionals caring for these infants during the neonatal stay and those in the post-discharge phase, including respiratory specialists.

### Trend of PND use

PND use to prevent BPD or aid extubation in premature infants born <32 weeks GA in England and Wales has nearly doubled over the last 11 years, demonstrating a fluctuating trend of PND use from a high-dose protracted course in the 1990s to reduced use in the 2000s [29], and finally to the current increased use. Despite this increasing trend, BPD rates continue to increase [30]. This may reflect the difficulty in balancing the risk–benefit of PND use and the lack of evidence in determining the ideal timing of using PND in high-risk infants. In 2020, PND was typically commenced >2 weeks later than the earliest age suggested by national guidance [10], with a further 34% of infants requiring repeated PND courses.

### Timing of commencement of PND

Our study suggests that PND commenced between 8 and 35 days chronological age was associated with a lower incidence of severe BPD, extubation at an earlier PMA and potentially lower need for respiratory support at discharge. These findings persisted after minimising the lead time, survival and residual bias (see Strengths and limitations) *via* the sensitivity analyses performed. This suggests that the anti-inflammatory effect of PND may become less effective the later PND is commenced, possibly reflecting more severe lung injury secondary to prolonged ventilation, which is known to be a good predictor of severe BPD during the first month of life [31] and the generalised pro-inflammatory state of premature infants.

The higher odds of death before discharge with earlier PND use cannot be fully explored with the present study methodology. This may be partly explained by the selection bias of infants receiving PND earlier that was not accounted for in our modelling, rather than a true casual effect. The association was not seen in the sensitivity analysis to minimise survival bias. This may explain the competing findings of the association found between later PND use with higher odds of severe BPD and/or death but lower mortality odds. Acutely unwell infants are more likely to receive dexamethasone earlier while infants who received dexamethasone later had already survived longer by definition. A previous meta-analysis found that the commencement of postnatal corticosteroids after 7 days of age demonstrated a trend towards a reduction in mortality without significant impact on long-term neurodevelopmental outcomes [7].

Our findings were consistent with those found by Harmon *et al.* [32] and Cuna *et al.* [33]. Harmon *et al.* [32] concluded that postnatal corticosteroids should be considered before 50 days old for the lowest associated odds of severe BPD in their National Institute of Child Health and Human Development Neonatal Research Network cohort of 951 infants born between 2006 and 2012. The later age of 50 days suggested may be partly explained by the combination of the different postnatal corticosteroid types (dexamethasone and hydrocortisone) used in their study which may have different anti-inflammatory effects. Cuna *et al.* [33] found that delayed commencement of PND at 29–42 *versus* 14–28 days old was associated with worse short-term outcomes, including longer duration of invasive ventilation and oxygen requirement, although this was a single-centre cohort of just 55 infants born between 2011 and 2016.

A recent network meta-analysis found that moderate PND dose given at 8–14 days was superior to 13 other BPD preventative regimes, including a range of PND doses given later at 14–28 days, although the evidence was of low certainty and included only two studies recruiting 117 infants within the last 20 years (*i.e.* 2006–2010 and 2012–2013). Furthermore, the authors concluded that the top three most beneficial BPD preventative regimes were moderate-dose and high-dose PND given at 8–14 days as well as high-dose PND given at 14–28 days [12]. Previous randomised controlled trials comparing the different timing of commencing PND found no difference in respiratory outcomes at 36 weeks PMA between PND use at 7 *versus* 14 days [34, 35] and 2 *versus* 4 weeks [36], respectively. However, these trials were undertaken in the 1990s, whereby neonatal respiratory practices have changed significantly since with increased surfactant use and lung injury minimisation ventilation strategies.

### Strengths and limitations

The key strengths of this study include the large population with point-of-care data collected and the true reflection of contemporary practice with babies discharged as late as 2021. Due to the retrospective nature of the study, causation cannot be drawn from the associations seen. While *a priori* factors from the literature [10, 25] were accounted for in our propensity score estimation, including birth years to minimise confounding by changes in practices over time, there may be further unmeasured confounders, including confounding by indication and lead time bias due to different follow-up periods in the three PND groups before BPD is diagnosed at 36 weeks PMA. However, sensitivity analyses to minimise these biases revealed similar results, supporting the main study findings. Although neurodevelopmental outcomes were not available, previous meta-analyses [7] and observational studies [32, 37] found that the risk for neurodevelopmental impairment did not differ significantly by the chronological age of dexamethasone exposure after 7 days old. We did, however, choose severe BPD as an outcome as this is known to be associated with worse neurodevelopmental outcomes [4] and preterm infants discharged from hospital on respiratory support have significantly more respiratory morbidity compared with those discharged without respiratory support [38].

Although the dose and indication of PND were not available, the definition of PND used was based on the current clinical practice [8], ensuring that the PND was intended to facilitate extubation and prevent BPD. Although the use of other postnatal corticosteroids may affect the results, this is unlikely to change the findings as dexamethasone remained the predominantly used postnatal corticosteroid for BPD [39]. Data inaccuracies and missing data could not be controlled for as data were entered at the point of care. However, the missing data rate was balanced across the three treatment groups after weighting. The study did not explore the potential impact of morbidities associated with poor respiratory outcomes [1], such as necrotising enterocolitis and treated retinopathy of prematurity, as it was beyond the study's aim. These morbidities often occurred after PND was commenced and it was difficult to ascertain with certainty the onset of these morbidities. Besides respiratory support requirement at discharge, further long-term respiratory outcomes that are important for parents [40], such as hospital readmissions and exercise limitation, were not available and warrant investigation in prospective studies.

### Conclusions

Clinicians are increasingly using PND to prevent BPD in high-risk preterm infants despite the lack of evidence on the optimal time to commence PND. Although our study suggests that the optimal window for PND use was between 8 and 35 days chronological age, which is in line with previous smaller studies, residual confounding and survival bias cannot be excluded. This highlights a need for both a definitive adequately powered clinical trial, including long-term outcomes, on the optimal timing for PND use, and objective measures to support the early identification of high-risk infants for timely PND treatment.

## Supplementary material

10.1183/13993003.00825-2023.Supp1 **Please note:** supplementary material is not edited by the Editorial Office, and is uploaded as it has been supplied by the author.
Supplementary material ERJ-00825-2023.Supplement

## Shareable PDF

10.1183/13993003.00825-2023.Shareable1 This one-page PDF can be shared freely online.
Shareable PDF ERJ-00825-2023.Shareable
