# Supplementary material for: Impact of postnatal dexamethasone timing on preterm mortality and bronchopulmonary dysplasia: a propensity score analysis
Source: Eur Respir J. 2023 Oct 19;62(4):2300825. doi: 10.1183/13993003.00825-2023 (PMC10586235; doi:10.1183/13993003.00825-2023)
Supplement: Supplementary file 1 [file ERJ-00825-2023.Supplement.pdf]

SUPPLEMENTARY MATERIAL

Supplementary Table 1 ICD-10 codes used to identify major congenital anomaly exclusions and the associated number of babies excluded. [1]Sum exceeds the total number of exclusions as some infants had more than one anomaly.

ICD-10 code	Anomaly	Number of infants excluded[1]
Q00	Anencephaly and similar malformations	0
Q01	Encephalocele and similar malformations	1
Q05	Spina bifida and similar malformations	0
Q20	Congenital malformations of cardiac chambers and connections	0
Q20.3	Transposition of great arteries	0
Q21.2	Atrioventricular septal defect (AVSD)	1
Q21.3	Tetralogy of Fallot	3
Q21.91	Single atrium	0
Q21.92	Single ventricle	0
Q22	Congenital malformations of pulmonary and tricuspid valves	28
Q23	Congenital malformations of aortic and mitral valves	12
Q23.4	Hypoplastic left heart	1
Q25.1	Coarctation of aorta	8
Q25.4	Other congenital malformations of aorta	1
Q25.5	Atresia of pulmonary artery	0
Q25.6	Stenosis of pulmonary artery (PS)	22
Q25.8	Other congenital malformations of great arteries	0
Q26.2	Total anomalous pulmonary venous connection (TAPVD)	1
Q30.0	Choanal atresia	3
Q32	Congenital malformations of trachea and bronchus	6
Q33.0	Congenital cystic lung	8
Q33.2	Sequestration of lung	1
Q33.3	Agenesis of lung	0
Q33.4	Congenital bronchiectasis	0
Q33.5	Ectopic tissue in lung	0
Q33.6	Hypoplasia and dysplasia of lung	2
Q34.0	Anomaly of pleura	0
Q34.1	Congenital cyst of mediastinum	0
Q34.8	Other specified congenital malformations of respiratory system	1
Q35/Q36/Q37	Cleft lip and/or palate	27
Q39	Oesophageal atresia	7
Q41	Congenital absence, atresia and stenosis of small intestine	0
Q42	Congenital absence, atresia and stenosis of large intestine	0
Q60.1	Bilateral renal agenesis	0
Q60.6	Potter's syndrome	0
Q61.1	Polycystic kidney, infantile type	0
Q61.2	Polycystic kidney, adult type	0
Q64.1	Exstrophy of urinary bladder	0
Q64.2	Posterior urethral valves (PUV)	1
Q64.5	Congenital absence of bladder and urethra	0
Q77.1	Thanatophoric short stature	1
Q79.0	Congenital diaphragmatic hernia	2
Q79.1	Eventration of diaphragmatic hernia	2
Q79.2	Exomphalos	10
Q79.3	Gastroschisis	0
Q90	Down's syndrome	5
Q91	Edwards' syndrome and Patau's syndrome	0

Supplementary Table 2 Definition of the variables and the associated data items extracted from the National Neonatal Research Database.

Treatment	
**Variable**	**Data Items**
Postnatal dexamethasone (PND)	**Definition**: Dexamethasone treatment for more than two consecutive days in ventilated infants between 8 days of chronological age and 36 weeks of postmenstrual age (PMA). Dexamethasone eye drops were excluded from this definition. This definition is based on current practices [1] to ensure that they are intended to treat or prevent BPD rather than for other reasons. A washout period of 7 days was used to define repeated courses.<br>• Data extracted from "DRUGSDAY" variable in the "DAILY" dataset.<br>• Dichotomous (Yes/No)
**A-priori variables used for propensity score estimation [2, 3]**	
**Variable**	**Data Items**
Gestational age	• Data extracted from "GESTATIONDAYS" and "GESTATIONWEEKS" variables in the "EPISODES" dataset.<br>• Continuous in days
Birthweight z score	**Definition**: Birthweight z score derived from the UK-WHO growth chart [4].<br>• Data extracted from "BIRTHWEIGHT", "GESTATIONDAYS", "GESTATIONWEEKS" and "GENDER" variables in the "EPISODES" dataset.<br>• Continuous
Sex	• Data extracted from "GENDER" variable in the "EPISODES" dataset.<br>• Dichotomous (Male/Female)
Multiple gestation	• Data extracted from "FETUSNUMBER" variable of more than 1 in the "EPISODES" dataset.<br>• Dichotomous (Yes/No)
Maternal antenatal corticosteroids course	**Definition**: Any maternal antenatal corticosteroids received before delivery<br>• Data extracted from "STEROIDSANTENATALCOURSES" variable in the "EPISODES" dataset.<br>• Dichotomous (Yes/No)
Prolonged rupture of membrane	**Definition**: Rupture of membrane for more than 18 hours.<br>• Data extracted from "ROMTIMEANON" variable or "Prolonged rupture membranes" response from the "PROBLEMSDURINGPREGNANCY" variable in the "EPISODES" dataset.<br>• Dichotomous (Yes/No)
Maternal chorioamnionitis	**Definition**: Clinically suspected maternal chorioamnionitis by the obstetrics team.<br>• Data extracted from "Chorioamnionitis" response from the "PROBLEMSDURINGPREGNANCY" variable in the "EPISODES" dataset as well as the "DIAGNOSISATADMISSION" and "PRINCIPALDIAGNOSISATDISCHARGE" variables in the "EPISODES" dataset for "Chorioamnionitis".<br>• Dichotomous (Yes/No)
Maternal gestational diabetes	• Data extracted from "Gestational diabetes" response from the "PROBLEMSDURINGPREGNANCY" variable in the "EPISODES" dataset.<br>• Dichotomous (Yes/No)

Maternal diabetes	• Data extracted from "Diabetes" response from the "PROBLEMSMEDICALMOTHER" variable in the "EPISODES" dataset.<br>• Dichotomous (Yes/No)
Maternal pre-eclampsia	• Data extracted from "Pregnancy induced hypertension", "Pre-eclampsia" and "Maternal HELLP" responses from the "PROBLEMSDURINGPREGNANCY" variable in the "EPISODES" dataset.<br>• Dichotomous (Yes/No)
Maternal hypertension	• Data extracted from "Chronic hypertension" response from the "PROBLEMSMEDICALMOTHER" variable in the "EPISODES" dataset.<br>• Dichotomous (Yes/No)
Maternal age	• Data extracted from "BIRTHYEARMOTHER" and "BIRTHYEAR" variables in the "EPISODES" dataset.<br>• Continuous
Level of care of neonatal unit at birth	• Data extracted from "POBNDAUCODE" variable in the "EPISODES" dataset and the "UNITLEVEL" variable in the "UNITLEVELSANDNDAUCODES" dataset.<br>• Categorical (1/2/3)
Neonatal network at birth	• Data extracted from "POBNDAUCODE" variable in the "EPISODES" dataset and the "NEONATAL_ODN" variable in the "ODNMAPPINGANDNDAUCODES" dataset.<br>• Categorical (14 networks)
Apgar score at 5 minutes	• Data extracted from "APGAR_5MIN" variable in the "EPISODES" dataset.<br>• Continuous
Cardiopulmonary resuscitation at birth in infants <30 weeks	**Definition**: Cardiopulmonary resuscitation provided at birth in infants born below 30 weeks of gestation<br>• Data extracted from "METHODSOFRESUSCITATION" variable in the "EPISODES" dataset if the "GESTATIONWEEKS" variables in the "EPISODES" dataset is <30.<br>• Dichotomous (Yes/No)
Admission temperature below 35°C	**Definition**: Admission temperature below 35°C on the <u>first</u> neonatal admission.<br>• Data extracted from "ADMITTEMPERATURE" variable from the "EPISODES" dataset if "EPISODENUMBERBABY" variable in the "EPISODES" dataset is 1.<br>• Dichotomous (Yes/No)
Inotrope requirement in the first week of life	**Definition**: Inotrope (Dopamine, dobutamine, adrenaline, noradrenaline and milrinone) requirement in the first week of life.<br>• Data extracted from "DRUGSDAY" and "INOTROPESGIVEN" variables from the "DAILY" dataset in the first week of age.<br>• Dichotomous (Yes/No)
Invasive ventilation in the first week of life	**Definition**: Invasive ventilation in the first week of life.<br>• Data extracted from "RESPIRATORYSUPPORT", "ADDEDO2", "VENTILATIONMODE" and "NONINVASIVERESPIRATORYSUPPORT" variables from the "DAILY" dataset in the first week of age.<br>• Dichotomous (Yes/No)
Clinical sepsis with or without positive blood or CSF culture in the first week of life	**Definition**: Clinically suspected sepsis receiving at least five consecutive days of antibiotics (Benzylpenicillin, amoxicillin, flucloxacillin, gentamicin, metronidazole, meropenem, cefotaxime, ceftazidime, cefradine, ceftriaxone and vancomycin)

	or blood/CSF culture that grew organism of clear pathogenicity as defined by the National Neonatal Audit Programme 2021 [5] in the first week of age.<br>• Data extracted from “DRUGSDAY” variable in the “DAILY” dataset as well as “PATHOGENSBLOODFORDBSYNCS” and “PATHOGENSCSFFORDBSYNCS” variables in the “SEPSIS SCREENS” dataset.<br>• Dichotomous (Yes/No)
Enteral feed type in the first week of life	• Data extracted from “DAYENTERALFEEDS” and “FORMULANAME” variables in the “DAILY” dataset in the first week of age.<br>• Categorical (Breast milk only, formula milk only, mixed formula and breast milk, nil by mouth)
Surfactant treatment	**Definition**: Surfactant treatment received at resuscitation or after admission to the neonatal unit.<br>• Data extracted from “SURFACTANTGIVENRESUSCITATION” variable in the “EPISODES” dataset and “DAYSURFACTANTGIVEN” and “DRUGSDAY” variables in the “DAILY” dataset.<br>• Dichotomous (Yes/No)
Birth year	• Data extracted from BIRTHYEAR” variable in the “EPISODES” dataset.<br>• Continuous
**Other variable**	
**Variable**	**Data Items**
NMR-2000 score	**Definition**: Neonatal mortality risk score derived from birthweight, highest level of respiratory support at any point within 24 hours of birth and oxygen saturation on admission [6].<br>• Data extracted from “BIRTHWEIGHT” and “ADMISSIONOXYGENSATURATION” variables in the “EPISODES” dataset as well as from “RESPIRATORYSUPPORT”, “ADDEDO2”, “VENTILATIONMODE” and “NONINVASIVERESPIRATORYSUPPORT” variables from the “DAILY” dataset on day 1 of age.<br>• Continuous
**Outcome**	
**Variable**	**Data Items**
Death	**Definition**: Death before discharge from the neonatal unit.<br>• Data extracted from “DATEOFDEATH” and “DISCHARGEDESTINATION” variables from the “EPISODES” dataset.<br>• Dichotomous (Yes/No)
Bronchopulmonary dysplasia (BPD)	**Definition**: Respiratory support or oxygen requirement over a three-day period at 36 weeks PMA or at discharge (if discharged before 36 weeks PMA [7, 8]).<br>• Data extracted from “RESPIRATORYSUPPORT”, “ADDEDO2”, “VENTILATIONMODE” and “NONINVASIVERESPIRATORYSUPPORT” variables from the “DAILY” dataset over a three-day period at 36 weeks PMA or at discharge.<br>• Dichotomous (Yes/No)
Severe BPD	**Definition**: Non-invasive (including high flow) and invasive ventilation requirement at 36 weeks PMA or at discharge (if discharged before 36 weeks PMA [7, 8]).

	• Data extracted from "RESPIRATORYSUPPORT", "ADDEDO2", "VENTILATIONMODE" and "NONINVASIVERESPIRATORYSUPPORT" variables from the "DAILY" dataset over a three-day period at 36 weeks PMA or at discharge.<br>• Dichotomous (Yes/No)
Respiratory support at discharge	**Definition**: Respiratory support or oxygen requirement at discharge.<br>• Data extracted from "RESPIRATORYSUPPORT", "ADDEDO2", "VENTILATIONMODE" and "NONINVASIVERESPIRATORYSUPPORT" variables from the "DAILY" dataset at discharge.<br>• Dichotomous (Yes/No)
Days of invasive ventilation	**Definition**: Duration of invasive ventilation in days<br>• Data extracted from "RESPIRATORYSUPPORT", "ADDEDO2", "VENTILATIONMODE" and "NONINVASIVERESPIRATORYSUPPORT" variables from the "DAILY" dataset.<br>• Continuous in days
Successful extubation within 14 days of starting postnatal dexamethasone	**Definition**: Successfully extubated for at least 7 days within 14 days after the start of dexamethasone course<br>• Data extracted from "RESPIRATORYSUPPORT", "ADDEDO2", "VENTILATIONMODE" and "NONINVASIVERESPIRATORYSUPPORT" variables from the "DAILY" dataset.<br>• Dichotomous (Yes/No)
Postmenstrual age when successfully extubated after receiving postnatal dexamethasone	**Definition**: PMA when successfully extubated for at least 7 days after the dexamethasone course<br>• Data extracted from "GESTATIONDAYS" and "GESTATIONWEEKS" variables in the "EPISODES" dataset as well as "RESPIRATORYSUPPORT", "ADDEDO2", "VENTILATIONMODE" and "NONINVASIVERESPIRATORYSUPPORT" variables from the "DAILY" dataset.<br>• Continuous in weeks

References

1. Job S, Clarke P. Current UK practices in steroid treatment of chronic lung disease. *Arch Dis Child Fetal Neonatal Ed* 2015: 100(4): F371.
2. National Institute for Health and Care Excellence. Specialist neonatal respiratory care for babies born preterm (NG124). NICE 2019.
3. British Association of Perinatal Medicine. Perinatal Management of Extreme Preterm Birth before 27 weeks of gestation. A Framework for Practice. October 2019. https://www.bapm.org/resources/80-perinatal-management-of-extreme-preterm-birth-before-27-weeks-of-gestation-2019 . [Date last accessed: October 12 2020].
4. Royal College of Paediatrics and Child Health. UK-WHO growth charts - neonatal and infant close monitoring (NICM). https://www.rcpch.ac.uk/resources/uk-who-growth-charts-neonatal-infant-close-monitoring-nicm. Date last accessed: March 01 2021.
5. National Neonatal Audit Programme (NNAP) Project Board. National Neonatal Audit Programme Annual Report 2021 - on 2020 data. Royal College of Paediatrics and Child Health. March 2022.
6. Medvedev MM, Brotherton H, Gai A, Tann C, Gale C, Waiswa P, Elbourne D, Lawn JE, Allen E. Development and validation of a simplified score to predict neonatal mortality risk among neonates

weighing 2000 g or less (NMR-2000): an analysis using data from the UK and The Gambia. *The Lancet Child & Adolescent Health* 2020: 4(4): 299-311.
7. Higgins RD, Jobe AH, Koso-Thomas M, Bancalari E, Viscardi RM, Hartert TV, Ryan RM, Kallapur SG, Steinhorn RH, Konduri GG, Davis SD, Thebaud B, Clyman RI, Collaco JM, Martin CR, Woods JC, Finer NN, Raju TNK. Bronchopulmonary Dysplasia: Executive Summary of a Workshop. *J Pediatr* 2018: 197: 300-308.
8. National Neonatal Audit Programme (NNAP) Project Board. National Neonatal Audit Programme Annual Report 2018 - on 2017 data. Royal College of Paediatrics and Child Health. September 2018.

Supplementary Table 3 Missing cases for the a-priori variables and outcomes used in the propensity score analysis.

Characteristics	Number of missing cases, n (%)
**A-priori variables**	
Birthweight Z score	33 (0.9)
Maternal antenatal corticosteroids	17 (0.5)
Maternal age	10 (0.3)
Admission temperature	86 (2.4)
Ventilated in the first 7 days of age	2 (0.1)
Inotrope requirement in the first 7 days of age	2 (0.1)
Enteral feed in the first 7 days of age	2 (0.1)
Apgar at 5 minutes	499 (14.1)
Neonatal network at birth	58 (1.6)
**Outcomes**	
Respiratory support at discharge	17 (0.5)

Supplementary Table 1 Characteristics of infants in the overall cohort (N=84,440), unweighted (N=3,469) and weighted infant cohort (effective sample size (ES)=3,032) depending on chronological age when infants received postnatal dexamethasone (two to three weeks ($PND^{2/3}$); four to five weeks ($PND^{4/5}$); and after six weeks (PND^{6+})). IQR = interquartile range.

Characteristics	Overall cohort (n=84,440)	Chronological age when postnatal dexamethasone was commenced (n=3,469)						
		Unweighted cohort				Weighted cohort[1]		
		$PND^{2/3}$ (N = 1,255)	$PND^{4/5}$ (N = 1,371)	PND^{6+} (N = 843)	p value[2]	$PND^{2/3}$ (ES = 1,098)	$PND^{4/5}$ (ES = 1,298)	PND^{6+} (ES = 636)
**<u>Infant factors at birth</u>**								
Gestational age (weeks), median (IQR)	29^{+3} (27^{+2} – 30^{+6})	25^{+1} (24^{+1} – 26^{+3})	25^{+1} (24^{+2} – 26^{+2})	24^{+5} (24^{+0} – 26^{+0})	<0.001	25^{+0} (24^{+1} – 26^{+2})	25^{+0} (24^{+1} – 26^{+1})	25^{+0} (24^{+1} – 26^{+1})
Missing data, n (%)	2 (0.0)	0 (0)	0 (0)	0 (0)		0	0	0
Birthweight (g), median (IQR)	1,197 (900 – 1,490)	710 (610 – 830)	707 (615 – 820)	670 (590 – 780)	<0.001	700 (610 – 817)	700 (610 – 815)	691 (601 – 805)
Missing data, n (%)	11 (0.0)	0 (0)	0 (0)	0 (0)		0	0	0
Birthweight Z score, median (IQR)	-0.26 (-0.88 – 0.26)	-0.56 (-1.17 – -0.06)	-0.58 (-1.18 – -0.11)	-0.63 (-1.18 – -0.19)	0.035	-0.57 (-1.17 – -0.11)	-0.59 (-1.18 – -0.11)	-0.59 (-1.16 – -0.12)
Missing data, n (%)	314 (0.4)	11 (0.9)	10 (0.7)	12 (1.4)		0.8	1.0	0.8
Male, n (%)	46,118 (54.6)	764 (60.9)	823 (60.0)	490 (58.1)	0.447	60.8	59.8	58.5
Missing data	62 (0.1)	0 (0)	0 (0)	0 (0)		0	0	0
Birth year, n (%)								
2010	7,519 (8.9)	59 (4.7)	83 (6.1)	71 (7.9)	0.002	5.1	6.1	6.6
2011	7,848 (9.3)	95 (7.6)	104 (7.6)	60 (7.1)		7.8	7.9	7.7
2012	7,915 (9.4)	108 (8.6)	98 (7.1)	89 (10.6)		8.2	7.4	8.7
2013	7,873 (9.3)	118 (9.4)	111 (8.1)	76 (9.0)		9.3	8.4	8.6
2014	7,765 (9.2)	99 (7.9)	125 (9.1)	67 (7.9)		8.4	8.6	8.0
2015	8,016 (9.5)	98 (7.8)	149 (10.9)	79 (9.4)		9.0	10.1	9.9
2016	8,064 (9.5)	113 (9.0)	145 (10.6)	75 (8.9)		10.0	9.9	9.3
2017	7,937 (9.4)	137 (10.9)	133 (9.7)	93 (11.0)		10.5	10.0	10.9
2018	7,396 (8.8)	149 (11.9)	139 (10.1)	87 (10.3)		10.9	10.5	11.3
2019	7,380 (8.7)	166 (13.2)	148 (10.8)	85 (10.1)		12.3	11.6	10.5
2020	6,727 (8.0)	113 (9.0)	136 (9.9)	61 (7.2)		8.5	9.5	8.6
Multiple pregnancy, n (%)	22,130 (26.2)	271 (21.6)	330 (24.1)	213 (25.3)	0.119	21.8	23.4	24.5
Missing data	22 (0.0)	0 (0)	0 (0)	0 (0)		0	0	0

Antenatal corticosteroids, n (%)	75,637 (89.6)	1,105 (88.0)	1,265 (92.3)	755 (89.6)	0.001	89.9	90.5	91.1
Missing data	715 (0.8)	3 (0.2)	9 (0.7)	5 (0.6)		0.3	0.5	0.5
**Maternal factors**								
Prolonged rupture of membrane, n (%)	9,727 (11.5)	158 (12.6)	199 (14.5)	98 (11.6)	0.116	12.6	13.3	11.5
Chorioamnionitis, n (%)	3,534 (4.2)	81 (6.5)	93 (6.8)	60 (7.1)	0.836	7.0	6.7	6.7
Maternal diabetes, n (%)	1,528 (1.8)	15 (1.2)	24 (1.8)	10 (1.2)	0.394	1.4	1.5	1.5
Gestational diabetes, n (%)	3,064 (3.6)	11 (0.9)	26 (1.9)	13 (1.5)	0.087	0.6	1.6	1.4
Maternal hypertension, n (%)	1,145 (1.4)	20 (1.6)	35 (2.6)	16 (1.9)	0.209	1.9	2.2	1.5
Gestational hypertension, n (%)	9,377 (11.1)	134 (10.7)	148 (10.8)	82 (9.7)	0.703	11.3	10.7	10.0
Maternal age (years), median (IQR)	31 (26 – 35)	30 (26 – 35)	30 (26 – 35)	30 (26 – 34)	0.669	30 (26 – 35)	30 (26 – 35)	30 (26 – 34)
Missing data, n (%)	584 (0.7)	3 (0.2)	2 (0.1)	4 (0.5)		0.4	0.2	0.5
**Infant factors after birth**								
Cardiopulmonary resuscitation at birth, n (%)	2,826 (3.3)	92 (7.3)	97 (7.1)	48 (5.7)	0.311	6.8	6.9	6.1
5 minute Apgar score, median (IQR)	8 (7 – 9)	7 (5 – 8)	7 (5 – 8)	7 (5 – 8)	0.095	7 (5 – 8)	7 (5 – 8)	7 (5 – 8)
Missing data, n (%)	9,286 (11.0)	209 (16.7)	197 (14.4)	90 (10.7)		14.6	13.7	12.7
Admission temperature <35ºC, n (%)	1,455 (1.7)	58 (4.6)	40 (2.9)	42 (5.0)	0.034	4.0	3.6	4.1
Missing data	1,040 (1.2)	35 (2.8)	37 (2.7)	14 (1.7)		2.5	2.5	1.6
Invasive ventilation in first 7 days, n (%)	53,845 (63.8)	1,252 (99.8)	1,357 (99.0)	837 (99.3)	0.146	99.7	99.3	99.3
Missing data	393 (0.5)	0 (0.0)	1 (0.1)	1 (0.1)		0.0	0.1	0.1
Inotrope use in first 7 days, n (%)	15,949 (18.9)	721 (57.5)	760 (55.4)	490 (58.1)	0.527	57.2	56.7	55.9
Missing data	393 (0.5)	0 (0.0)	1 (0.1)	1 (0.1)		0.0	0.1	0.1
Sepsis in first 7 days, n (%)	33,993 (40.3)	897 (71.5)	983 (71.7)	652 (77.3)	0.005	72.7	72.7	73.9
Feed type in first 7 days, n (%)								
Nil by mouth	6,139 (7.3)	118 (9.4)	159 (11.6)	113 (12.5)	0.008	9.9	11.4	12.2
Breast milk only	54,599 (64.7)	1,095 (87.3)	1,132 (82.6)	704 (83.5)		86.2	84.2	83.9
Mixed breast and formula milk	18,332 (21.7)	34 (2.7)	62 (4.5)	21 (2.5)		3.2	3.3	2.9
Formula milk only	3,713 (4.4)	8 (0.6)	17 (1.2)	8 (0.9)		0.8	1.0	0.9
Missing data	1,657 (2.0)	0 (0.0)	1 (0.1)	1 (0.1)		0.0	0.1	0.1
Surfactant use, n (%)	52,225 (61.8)	1,216 (96.9)	1,338 (97.6)	806 (95.6)	0.034	96.9	97.1	96.6
NMR-2000 score [1], median (IQR)	10 (7 – 14)	5 (4 – 7)	5 (4 – 7)	5 (4 – 6)	<0.0001	5 (4 – 6)	5 (4 – 7)	5 (4 – 6)
Missing data, n (%)	9,281 (11.0)	129 (10.3)	129 (9.4)	90 (10.7)		10.1	9.4	11.0

Mortality risk based on NMR-2000 [1], n (%)								
Low risk	10,785 (12.8)	3 (0.2)	2 (0.1)	1 (0.1)		0.2	0.1	0.2
Moderate risk	54,747 (64.8)	515 (41.0)	588 (42.9)	295 (35.0)	0.021	40.3	41.3	38.4
High risk	9,627 (11.4)	608 (48.4)	652 (47.6)	457 (54.2)		49.4	49.2	50.4
Missing data	9,281 (11.0)	129 (10.3)	129 (9.4)	90 (10.7)		10.1	9.4	11.0
Organisational factor								
Neonatal unit level at birth, n (%)								
Level 1	6,395 (7.6)	58 (4.6)	78 (5.7)	56 (6.6)	0.306	4.7	5.6	5.5
Level 2	31,433 (37.2)	258 (20.6)	267 (19.5)	157 (18.6)		19.7	19.6	20.1
Level 3	46,595 (55.2)	939 (74.8)	1,026 (74.8)	630 (74.7)		75.5	74.8	74.3
Missing data	17 (0)	0 (0)	0 (0)	0 (0)		0	0	0
Neonatal network at birth, n (%)	82,955 (98.2)							
% spread across networks, median (IQR)	N/A	6.3 (3.6 – 9.8)	6.8 (5.2 – 9.3)	6.8 (4.5 – 9.8)	<0.001	5.9 (4.4 – 8.9)	6.6 (4.7 – 9.3)	6.7 (5.2 – 9.5)
Missing data	1,485 (1.8)	26 (2.1)	18 (1.3)	13 (1.5)		1.6	1.5	1.4

[1] Only the percentage of infants with the corresponding categorical variables was depicted for the weighted infant cohort.

[2] Chi-squared test and Kruskal-Wallis rank sum test were used to compare the categorical and continuous infant characteristics respectively across the three postnatal dexamethasone treatment groups.

References

1. Medvedev MM, Brotherton H, Gai A, Tann C, Gale C, Waiswa P, Elbourne D, Lawn JE, Allen E. Development and validation of a simplified score to predict neonatal mortality risk among neonates weighing 2000 g or less (NMR-2000): an analysis using data from the UK and The Gambia. *The Lancet Child & Adolescent Health* 2020: 4(4): 299-311.

Supplementary Table 5 Neonatal outcomes between infants who received postnatal dexamethasone (PND) at the chronological age of two to three weeks ($PND^{2/3}$); four to five weeks ($PND^{4/5}$); and after six weeks (PND^{6+}) in the unweighted and weighted infant cohort after excluding infants who received PND after 32 weeks of postmenstrual age (PMA) (N=2,986). n = number of infants. OR = Odds ratio. MD = Mean difference. CI = confidence interval. IQR = interquartile range. * = statistically significant after Bonferroni correction.

Characteristics	Chronological age when PND was commenced excluding PND >32 weeks PMA (N=2,986)							
	Unweighted cohort			Weighted cohort[1]				
	$PND^{2/3}$ (n = 1,239)	$PND^{4/5}$ (n = 1,275)	PND^{6+} (n = 472)	$PND^{2/3}$	$PND^{4/5}$	PND^{6+}	Treatment effect OR/MD (95% CI)	p value
**Primary outcome**								
Severe bronchopulmonary dysplasia and/or death, n (%)	979 (79.0)	1014 (79.5)	399 (84.5)	79.6	79.2	84.8	$PND^{2/3}$: Reference<br>$PND^{4/5}$: 0.98 (0.80 – 1.20)<br>PND^{6+}: 1.46 (1.06 – 2.03)	 0.868<br>0.022
**Secondary outcomes**								
Death, n (%)	270 (21.8)	158 (12.4)	44 (9.3)	21.7	12.7	8.2	$PND^{2/3}$: Reference<br>$PND^{4/5}$: 0.52 (0.41 – 0.65)<br>PND^{6+}: 0.30 (0.21 – 0.44)	 <0.001*<br><0.001*
Severe bronchopulmonary dysplasia, n (%)[2]	709 (73.2)	856 (76.6)	355 (82.9)	73.9	76.2	83.4	$PND^{2/3}$: Reference<br>$PND^{4/5}$: 1.14 (0.93 – 1.41)<br>PND^{6+}: 1.85 (1.33 – 2.59)	 0.211<br><0.001*
Bronchopulmonary dysplasia, n (%)[2]	917 (94.6)	1067 (95.5)	416 (97.2)	94.9	95.3	96.8	$PND^{2/3}$: Reference<br>$PND^{4/5}$: 1.09 (0.72 – 1.64)<br>PND^{6+}: 1.59 (0.76 – 3.32)	 0.682<br>0.219
Duration of invasive ventilation (days), median (IQR)[2]	30 (22 – 44)	35 (28 – 45)	49 (41 – 58)	31 (22 – 45)	35 (28 – 46)	46 (40 – 55)	$PND^{2/3}$: Reference<br>$PND^{4/5}$: 3.3 (1.7 – 5.0)<br>PND^{6+}: 12.2 (10.1 – 14.4)	 <0.001*<br><0.001*
Successful extubation within 14 days of starting PND, n (%)[2]	598 (61.7)	838 (75.0)	339 (79.2)	58.8	74.4	79.9	$PND^{2/3}$: Reference<br>$PND^{4/5}$: 2.3 (2.0 – 2.8)<br>PND^{6+}: 3.8 (2.9 – 5.1)	 <0.001*<br><0.001*
PMA when successfully extubated after receiving PND (weeks), median (IQR)[2]	29^{+5} (28^{+3} – 31^{+2})	30^{+3} (29^{+2} – 31^{+5})	31^{+4} (30^{+5} – 32^{+3})	29^{+4} (28^{+3} – 31^{+2})	30^{+2} (29^{+2} – 31^{+4})	31^{+5} (30^{+6} – 32^{+4})	$PND^{2/3}$: Reference<br>$PND^{4/5}$: 0.7 (0.5 – 0.9)<br>PND^{6+}: 2.3 (2.0 – 2.5)	 <0.001*<br><0.001*
Respiratory support at discharge, n (%)[2]	649 (67.0)	750 (67.1)	313 (73.1)	67.3	67.3	75.4	$PND^{2/3}$: Reference<br>$PND^{4/5}$: 1.01 (0.84 – 1.23)<br>PND^{6+}: 1.52 (1.13 – 2.05)	 0.896<br>0.005*

[1] Only the percentage of infants with the respective categorical neonatal outcomes was depicted for the weighted infant cohort.

[2] Infants who died before discharge were excluded from the analysis.

Supplementary Table 6 Neonatal outcomes between infants who received postnatal dexamethasone (PND) at the chronological age of two to three weeks ($PND^{2/3}$); four to five weeks ($PND^{4/5}$); and after six weeks (PND^{6+}) in the unweighted and weighted infant cohort after excluding infants who received PND after 32 weeks of postmenstrual age (PMA) or infants who died before 6 weeks old (N=2,760). N = number of infants. OR = Odds ratio. MD = Mean difference. CI = confidence interval. IQR = interquartile range. * = statistically significant after Bonferroni correction.

**Characteristics**	**Chronological age when PND was commenced excluding PND >32 weeks PMA or died <6 weeks old (N=2,760)**							
	**Unweighted cohort**			**Weighted cohort**[1]				
	**$PND^{2/3}$** (n = 1,070)	**$PND^{4/5}$** (n = 1,220)	**PND^{6+}** (n = 470)	**$PND^{2/3}$**	**$PND^{4/5}$**	**PND^{6+}**	**Treatment effect OR/MD (95% CI)**	**p value**
**<u>Primary outcome</u>**								
Severe bronchopulmonary dysplasia and/or death, n (%)	810 (75.7)	959 (78.6)	397 (84.5)	76.2	78.2	84.7	$PND^{2/3}$: Reference<br>$PND^{4/5}$: 1.13 (0.92 – 1.39)<br>PND^{6+}: 1.79 (1.29 – 2.48)	 0.238<br><0.001*
**<u>Secondary outcomes</u>**								
Death, n (%)	101 (9.4)	103 (8.4)	42 (8.9)	9.2	8.6	7.5	$PND^{2/3}$: Reference<br>$PND^{4/5}$: 0.92 (0.68 – 1.24)<br>PND^{6+}:0.77 (0.51 – 1.17)	 0.582<br>0.227
Severe bronchopulmonary dysplasia, n (%)[2]	709 (73.2)	856 (76.6)	355 (82.9)	73.8	76.2	83.4	$PND^{2/3}$: Reference<br>$PND^{4/5}$: 1.15 (0.93 – 1.41)<br>PND^{6+}: 1.86 (1.34 – 2.59)	 0.200<br><0.001*
Bronchopulmonary dysplasia, n (%)[2]	917 (94.6)	1067 (95.5)	416 (97.2)	94.9	95.3	96.8	$PND^{2/3}$: Reference<br>$PND^{4/5}$: 1.09 (0.72 – 1.66)<br>PND^{6+}: 1.60 (0.77 – 3.34)	 0.668<br>0.207
Duration of invasive ventilation (days), median (IQR)[2]	30 (22 – 44)	35 (28 – 45)	49 (41 – 58)	31 (22 – 45)	35 (28 – 46)	46 (40 – 55)	$PND^{2/3}$: Reference<br>$PND^{4/5}$: 3.4 (1.7 – 5.0)<br>PND^{6+}: 12.4 (10.3 – 14.5)	 <0.001*<br><0.001*
Successful extubation within 14 days of starting PND, n (%)[2]	598 (61.7)	838 (75.0)	339 (79.2)	58.4	74.4	79.8	$PND^{2/3}$: Reference<br>$PND^{4/5}$: 2.2 (1.8 – 2.7)<br>PND^{6+}: 3.4 (2.4 – 4.8)	 <0.001*<br><0.001*
PMA when successfully extubated after receiving PND (weeks), median (IQR)[2]	29^{+5}<br>(28^{+3} – 31^{+2})	30^{+3}<br>(29^{+1}2– 31^{+5})	31^{+4}<br>(30^{+5} – 32^{+3})	29^{+4}<br>(28^{+3} – 31^{+2})	30^{+2}<br>(29^{+2} – 31^{+4})	31^{+5}<br>(30^{+6} – 32^{+4})	$PND^{2/3}$: Reference<br>$PND^{4/5}$: 0.9 (0.7 – 1.1)<br>PND^{6+}: 2.6 (2.4 – 2.9)	 <0.001*<br><0.001*
Respiratory support at discharge, n (%)[2]	649 (67.0)	750 (67.1)	313 (73.1)	67.0	67.6	76.1	$PND^{2/3}$: Reference<br>$PND^{4/5}$: 1.04 (0.86 – 1.26)<br>PND^{6+}: 1.60 (1.20 – 2.15)	 0.672<br>0.002*

[1] Only the percentage of infants with the respective categorical neonatal outcomes was depicted for the weighted infant cohort.

[2] Infants who died before discharge were excluded from the analysis.

Supplementary Table 7 Neonatal outcomes between infants who received postnatal dexamethasone (PND) at the chronological age of two to three weeks ($PND^{2/3}$); four to five weeks ($PND^{4/5}$); and after six weeks (PND^{6+}) in the unweighted and weighted infant cohort after excluding infants with propensity scores outside the 5th – 95th centile range (N=2,564). n = number of infants. OR = Odds ratio. MD = Mean difference. CI = confidence interval. IQR = interquartile range. PMA = postmenstrual age. * = statistically significant after Bonferroni correction.

Characteristics	Chronological age when PND was commenced excluding infants outside 5th – 95th centile range (N=2,564)							
	Unweighted cohort			Weighted cohort[1]				
	$PND^{2/3}$ (n = 925)	$PND^{4/5}$ (n = 1,233)	PND^{6+} (n = 406)	$PND^{2/3}$	$PND^{4/5}$	PND^{6+}	Treatment effect OR/MD (95% CI)	p value
**Primary outcome**								
Severe bronchopulmonary dysplasia and/or death, n (%)	733 (79.2)	986 (80.0)	353 (86.9)	79.6	79.8	86.1	$PND^{2/3}$: Reference<br>$PND^{4/5}$: 1.02 (0.82 – 1.26)<br>PND^{6+}: 1.58 (1.13 – 2.21)	 0.892<br>0.007
**Secondary outcomes**								
Death, n (%)	183 (19.8)	159 (12.9)	48 (11.8)	19.5	13.0	11.8	$PND^{2/3}$: Reference<br>$PND^{4/5}$: 0.62 (0.49 – 0.78)<br>PND^{6+}:0.55 (0.39 – 0.78)	 <0.001*<br><0.001*
Severe bronchopulmonary dysplasia, n (%)[2]	550 (74.1)	827 (77.0)	305 (85.2)	74.7	76.8	84.2	$PND^{2/3}$: Reference<br>$PND^{4/5}$: 1.12 (0.90 – 1.40)<br>PND^{6+}: 1.81 (1.29 – 2.54)	 0.298<br>0.001*
Bronchopulmonary dysplasia, n (%)[2]	704 (94.9)	1025 (95.4)	347 (96.9)	94.9	95.6	96.9	$PND^{2/3}$: Reference<br>$PND^{4/5}$: 1.15 (0.74 – 1.79)<br>PND^{6+}: 1.67 (0.83 – 3.35)	 0.536<br>0.152
Duration of invasive ventilation (days), median (IQR)[2]	30 (22 – 43)	35 (28 – 45)	52 (41 – 66)	30 (22 – 43)	35 (28 – 45)	51 (41 – 66)	$PND^{2/3}$: Reference<br>$PND^{4/5}$: 4.7 (3.0 – 6.5)<br>PND^{6+}: 22.6 (19.5 – 25.6)	 <0.001*<br><0.001*
Successful extubation within 14 days of starting PND, n (%)[2]	471 (63.5)	814 (75.8)	271 (75.7)	63.1	75.5	76.3	$PND^{2/3}$: Reference<br>$PND^{4/5}$: 1.8 (1.5 – 2.2)<br>PND^{6+}: 1.9 (1.4 – 2.5)	 <0.001*<br><0.001*
PMA when successfully extubated after receiving PND (weeks), median (IQR)[2]	29^{+5} (28^{+3} – 31^{+1})	30^{+4} (29^{+3} – 31^{+6})	32^{+4} (31^{+2} – 34^{+4})	29^{+5} (28^{+3} – 31^{+2})	30^{+4} (29^{+3} – 31^{+6})	32^{+4} (31^{+2} – 34^{+4})	$PND^{2/3}$: Reference<br>$PND^{4/5}$: 0.8 (0.6 – 1.0)<br>PND^{6+}: 3.2 (2.8 – 3.5)	 <0.001*<br><0.001*
Respiratory support at discharge, n (%)[2]	505 (68.1)	727 (67.7)	265 (74.0)	68.3	67.4	73.7	$PND^{2/3}$: Reference<br>$PND^{4/5}$: 0.97 (0.79 – 1.20)<br>PND^{6+}: 1.33 (1.00 – 1.78)	 0.802<br>0.053

[1] Only the percentage of infants with the respective categorical neonatal outcomes was depicted for the weighted infant cohort.

[2] Infants who died before discharge were excluded from the analysis.

Supplementary Table 8 Neonatal outcomes between infants who received postnatal dexamethasone (PND) for at least seven consecutive days at the chronological age of two to three weeks ($PND^{2/3}$); four to five weeks ($PND^{4/5}$); and after six weeks (PND^{6+}) (N=2,686). n = number of infants. OR = Odds ratio. MD = Mean difference. CI = confidence interval. IQR = interquartile range. PMA = postmenstrual age. * = statistically significant after Bonferroni correction.

**Characteristics**	**Chronological age when PND for at least 7 consecutive days was commenced (N=2,686)**							
	**Unweighted cohort**			**Weighted cohort**[1]				
	**$PND^{2/3}$**<br>(n = 894)	**$PND^{4/5}$**<br>(n = 1,073)	**PND^{6+}**<br>(n = 719)	**$PND^{2/3}$**	**$PND^{4/5}$**	**PND^{6+}**	**Treatment effect**<br>**OR/MD (95% CI)**	**p value**
**<u>Primary outcome</u>**								
Severe bronchopulmonary dysplasia and/or death, n (%)	711 (79.5)	866 (80.7)	635 (88.3)	80.3	80.4	87.5	$PND^{2/3}$: Reference<br>$PND^{4/5}$: 1.01 (0.80 – 1.27)<br>PND^{6+}: 1.73 (1.28 – 2.32)	 0.939<br><0.001*
**<u>Secondary outcomes</u>**								
Death, n (%)	150 (16.8)	121 (11.3)	73 (10.2)	16.6	11.7	10.2	$PND^{2/3}$: Reference<br>$PND^{4/5}$: 0.67 (0.51 – 0.87)<br>PND^{6+}:0.57 (0.41 – 0.79)	 0.003*<br>0.001*
Severe bronchopulmonary dysplasia, n (%)[2]	561 (75.4)	745 (78.3)	562 (87.0)	76.4	77.8	86.1	$PND^{2/3}$: Reference<br>$PND^{4/5}$: 1.09 (0.86 – 1.37)<br>PND^{6+}: 1.92 (1.42 – 2.60)	 0.486<br><0.001*
Bronchopulmonary dysplasia, n (%)[2]	712 (95.7)	916 (96.2)	631 (97.7)	96.0	96.1	97.4	$PND^{2/3}$: Reference<br>$PND^{4/5}$: 1.02 (0.62 – 1.68)<br>PND^{6+}: 1.55 (0.79 – 3.02)	 0.927<br>0.199
Duration of invasive ventilation (days), median (IQR)[2]	30 (22 – 44)	35 (28 – 44)	51 (42 – 64)	30 (22 – 45)	35 (28 – 44)	50 (41 – 61)	$PND^{2/3}$: Reference<br>$PND^{4/5}$: 2.9 (0.9 – 4.8)<br>PND^{6+}: 18.3 (15.9 – 20.7)	 <0.001*<br><0.001*
Successful extubation within 14 days of starting PND, n (%)[2]	467 (62.8)	752 (79.0)	515 (79.7)	61.5	78.5	80.4	$PND^{2/3}$: Reference<br>$PND^{4/5}$: 2.3 (1.8 – 2.9)<br>PND^{6+}: 2.6 (2.0 – 3.3)	 <0.001*<br><0.001*
PMA when successfully extubated after receiving PND (weeks), median (IQR)[2]	29^{+5}<br>(28^{+3} – 31^{+2})	30^{+3}<br>(29^{+2} – 31^{+5})	32^{+5}<br>(31^{+2} – 34^{+3})	29^{+5}<br>(28^{+3} – 31^{+2})	30^{+2}<br>(29^{+2} – 31^{+5})	32^{+5}<br>(31^{+2} – 34^{+3})	$PND^{2/3}$: Reference<br>$PND^{4/5}$: 0.6 (0.4 – 0.8)<br>PND^{6+}: 3.2 (2.9 – 3.5)	 <0.001*<br><0.001*
Respiratory support at discharge, n (%)[2]	515 (69.2)	673 (70.7)	495 (76.6)	69.6	70.4	76.1	$PND^{2/3}$: Reference<br>$PND^{4/5}$: 1.06 (0.85 – 1.32)<br>PND^{6+}: 1.41 (1.09 – 1.82)	 0.602<br>0.009

[1] Only the percentage of infants with the respective categorical neonatal outcomes was depicted for the weighted infant cohort.

[2] Infants who died before discharge were excluded from the analysis.

Supplementary Table 9 Participating neonatal units in England and Wales and their respective lead clinicians. The list was accessed from https://www.imperial.ac.uk/neonatal-data-analysis-unit/neonatal-data-analysis-unit/list-of-national-neonatal-units/ on 06/01/2022.

Institution	Lead clinician
Airedale General Hospital	Dr Matthew Babirecki
Arrowe Park Hospital	Dr Anand Kamalanathan
Barnet Hospital	Dr Tim Wickham
Barnsley District General Hospital	Dr Kavi Aucharaz
Basildon Hospital	Dr Aashish Gupta
Basingstoke & North Hampshire Hospital	Dr Nicola Paul
Bassetlaw District General Hospital	Dr L M Wong
Bedford Hospital	Dr Anita Mittal
Birmingham City Hospital	Dr Lindsay Halpern
Birmingham Heartlands Hospital	Dr Pinki Surana
Birmingham Women's Hospital	Dr Matt Nash
Bradford Royal Infirmary	Dr Sam Wallis
Broomfield Hospital, Chelmsford	Dr Ahmed Hassan
Calderdale Royal Hospital	Dr Karin Schwarz
Chelsea & Westminster Hospital	Dr Shu-Ling Chuang
Chesterfield & North Derbyshire Royal Hospital	Dr Aiwyne Foo
Colchester General Hospital	Dr Jo Anderson
Conquest Hospital	Dr Graham Whincup
Countess of Chester Hospital	Dr Stephen Brearey
Croydon University Hospital	Dr Morris
Croydon University Hospital	Dr Srirambhatla
Cumberland Infirmary	Dr Yee Aung
Darent Valley Hospital	Dr Abdul Hasib
Darlington Memorial Hospital	Dr Mehdi Garbash
Derriford Hospital	Dr Alex Allwood
Diana Princess of Wales Hospital	Dr Pauline Adiotomre
Doncaster Royal Infirmary	Dr Nigel Brooke
Dorset County Hospital	Dr Abby Deketelaere
East Surrey Hospital	Dr Abdul Khader
Epsom General Hospital	Dr Sonia Spathis
Frimley Park Hospital	Dr Sanghavi Rekha
Furness General Hospital	Dr Anas Olabi
George Eliot Hospital	Dr Mukta Jain
Glan Clwyd Hospital	Dr Ian Barnard
Glangwili General Hospital	Dr Prem Pitchaikani
Gloucester Royal Hospital	Dr Jennifer Holman
Good Hope Hospital	Dr Pinki Surana
Great Western Hospital	Dr Stanley Zengeya
Guy's & St Thomas' Hospital	Dr Geraint Lee
Harrogate District Hospital	Dr Sobia Balal

Hereford County Hospital	Dr Cath Seagrave
Hillingdon Hospital	Dr Tristan Bate
Hinchingbrooke Hospital	Dr Hilary Dixon
Homerton Hospital	Dr Narendra Aladangady
Hull Royal Infirmary	Dr Hassan Gaili
Ipswich Hospital	Dr Matthew James
James Cook University Hospital	Dr M Lal
James Paget Hospital	Dr Ambadkar
Kettering General Hospital	Dr Poornima Pandey
Kings College Hospital	Dr Ravindra Bhat
King's Mill Hospital	Dr Simon Rhodes
Kingston Hospital	Dr Jonathan Filkin
Lancashire Women and Newborn Centre	Dr Savi Sivashankar
Leeds Neonatal Service	Dr Lawrence Miall
Leicester General Hospital	Dr Jonathan Cusack
Leicester Royal Infirmary	Dr Venkatesh Kairamkonda
Leighton Hospital	Dr Michael Grosdenier
Lincoln County Hospital	Dr Ajay Reddy
Lister Hospital	Dr J Kefas
Liverpool Women's Hospital	Dr Christopher Dewhurst
Luton & Dunstable Hospital	Dr Jennifer Birch
Macclesfield District General Hospital	Dr Gail Whitehead
Manor Hospital	Dr Ashok Karupaiah
Medway Maritime Hospital	Dr Ghada Ramadan
Milton Keynes General Hospital	Dr I Misra
Musgrove Park Hospital	Dr Chris Knight
New Cross Hospital	Dr Matt Nash
Newham General Hospital	Dr Imdad Ali
Nobles Hospital	Dr Prakash Thiagarajan
Norfolk & Norwich University Hospital	Dr Muthukumar
North Devon District Hospital	Dr Michael Selter
North Manchester General Hospital	Dr Ajit Mahaveer
North Middlesex University Hospital	Dr Neeraj Jain
Northampton General Hospital	Dr Subodh Gupta
Northumbria Specialist Emergency Care Hospital	Jess Reynolds
Northwick Park Hospital	Dr Richard Nicholl
Nottingham City Hospital	Dr Steven Wardle
Nottingham University Hospital (QMC)	Dr Steven Wardle
Ormskirk District General Hospital	Dr Andreea Bontea
Oxford University Hospitals, John Radcliffe Hospital	Dr Eleri Adams
Peterborough City Hospital	Dr Katharine McDevitt
Pilgrim Hospital	Dr Ajay Reddy
Pinderfields General Hospital (Pontefract General Infirmary)	Dr David Gibson

Poole General Hospital	Prof Minesh Khashu
Prince Charles Hospital	Dr Iyad Al-Muzaffar
Princess Alexandra Hospital	Dr Chinnappa Reddy
Princess Anne Hospital	Dr Mark Johnson
Princess of Wales Hospital	Dr Kate Creese
Princess Royal Hospital	Dr P Amess
Princess Royal Hospital (previously Royal Shrewsbury Hospital)	Dr Deshpande
Princess Royal University Hospital	Dr Elizabeth Sleight
Queen Alexandra Hospital	Dr Charlotte Groves
Queen Charlotte's Hospital	Dr Lidia Tyszcuzk
Queen Elizabeth Hospital, Gateshead	Dr Anne Dale
Queen Elizabeth Hospital, King's Lynn	Dr Glynis Rewitzky
Queen Elizabeth Hospital, Woolwich - see notes	Dr Olutoyin Banjoko
Queen Elizabeth the Queen Mother Hospital	Dr Bushra Abdul-Malik
Queen's Hospital, Burton on Trent	Dr Dominic Muogbo
Queen's Hospital, Romford	Dr Khalid Mannan
Queen's Hospital, Romford 2	Dr Khalid Mannan
Rosie Maternity Hospital, Addenbrookes	Dr Angela D'Amore
Rotherham District General Hospital	Dr Soma Sengupta
Royal Albert Edward Infirmary	Dr Christos Zipitis
Royal Berkshire Hospital	Dr Peter De Halpert
Royal Bolton Hospital	Dr Paul Settle
Royal Cornwall Hospital	Dr Paul Munyard
Royal Derby Hospital	Dr John McIntyre
Royal Devon & Exeter Hospital	Dr Chrissie Oliver
Royal Gwent Hospital	Dr Sunil Reddy
Royal Hampshire County Hospital	Dr Lucinda Winckworth
Royal Lancaster Infirmary	Dr Joanne Fedee
Royal Oldham Hospital	Dr Natasha Maddock
Royal Preston Hospital	Dr Richa Gupta
Royal Stoke University Hospital	Dr Jyoti Kapur
Royal Surrey County Hospital	Dr Ben Obi
Royal Sussex County Hospital	Dr P Amess
Royal United Hospital	Dr Stephen Jones
Royal Victoria Infirmary	Dr Naveen Athiraman
Russells Hall Hospital	Dr Chandan Gupta
Salisbury District Hospital	Dr Jim Baird
Scarborough General Hospital	Dr Kirsten Mack
Scunthorpe General Hospital	Dr Pauline Adiotomre
Singleton Hospital	Dr Arun Ramachandran
Southend Hospital	Dr Vineet Gupta
Southmead Hospital	Dr Faith Emery
St George's Hospital	Dr Charlotte Huddy

St Helier Hospital	Dr Ralf Hartung
St Mary's Hospital, IOW	Dr Akinsola Ogundiya
St Mary's Hospital, London	Dr Lidia Tyszcuzk
St Mary's Hospital, Manchester	Dr Ngozi Edi-Osagie
St Michael's Hospital	Dr Pamela Cairns
St Peter's Hospital	Dr Peter Martin
St Richard's Hospital	Dr Victoria Sharp
Stepping Hill Hospital	Dr Carrie Heal
Stoke Mandeville Hospital	Dr Sanjay Salgia
Sunderland Royal Hospital	Dr Majd Abu-Harb
Tameside General Hospital	Dr Jacqeline Birch
The Grange University Hospital	Dr Sunil Reddy
The Jessop Wing, Sheffield	Dr Porus Bastani
The Royal Free Hospital	Dr Marice Theron
The Royal London Hospital - Constance Green	Dr Vadivelam Murthy
Torbay Hospital	Dr Siba Paul
Tunbridge Wells Hospital	Dr Hamudi Kisat
University College Hospital	Dr Giles Kendall
University Hospital Coventry	Dr Puneet Nath
University Hospital Lewisham	Dr Ozioma Obi
University Hospital of North Durham	Dr Mehdi Garbash
University Hospital of North Tees	Dr Hari Kumar
University Hospital of Wales	Dr Nitin Goel
Victoria Hospital, Blackpool	Dr Chris Rawlingson
Warrington Hospital	Dr Delyth Webb
Warwick Hospital	Dr Bird
Watford General Hospital	Dr Sankara Narayanan
West Cumberland Hospital	Dr Yee Aung
West Middlesex University Hospital	Dr Eleanor Hulse
West Suffolk Hospital	Dr Ian Evans
Wexham Park Hospital	Dr Sanjay Jaisal
Whipps Cross University Hospital	Dr Caroline Sullivan
Whiston Hospital	Dr Ros Garr
Whittington Hospital	Dr Wynne Leith
William Harvey Hospital	Dr Vimal Vasu
Withybush Hospital	Dr Vishwa Narayan
Worcestershire Royal Hospital	Dr Liza Harry
Worthing Hospital	Dr Katia Vamvakiti
Wrexham Maelor Hospital	Dr Brendan Harrington
Wythenshawe Hospital	Dr Ngozi Edi-Osagie
Yeovil District Hospital	Dr Megan Eaton
York District Hospital	Dr Sundeep Sandhu
Ysbyty Gwynedd	Dr Mike Cronin

Supplementary Figure 1: Balance plot demonstrating good balance for all the a-priori variables used in propensity score analysis amongst the three groups after weighting using the inverse probability of treatment weighted estimation with standardised mean differences and variance ratios ranging between 0.0007–0.05 and 1.01–1.17 respectively. BWZ = Birthweight Z scores, Miss = missing, ANS = Antenatal corticosteroids. PROM = Prolonged rupture of membrane. Mat = Maternal. Gest = Gestational. CPR = Cardiopulmonary resuscitation. Admit = Admission. Vent = ventilated. EBM = Expressed breast milk.

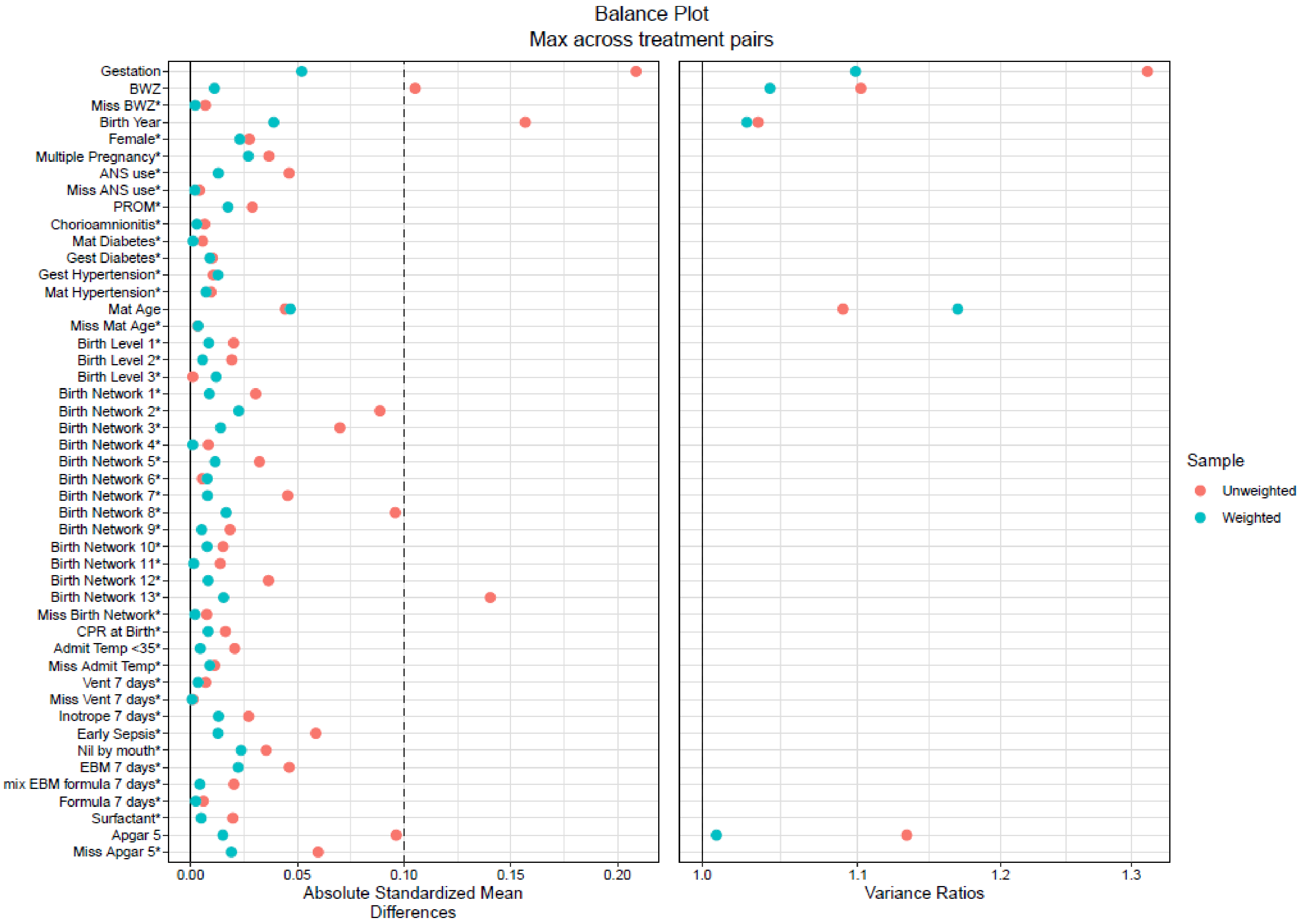

Supplementary Figure 2: Box plots of the estimated propensity scores for each of the three postnatal dexamethasone treatment (Tx) groups (2–3 weeks; 4–5 weeks; and ≥6 weeks) for every infant in the propensity score analysis cohort by the treatment groups.

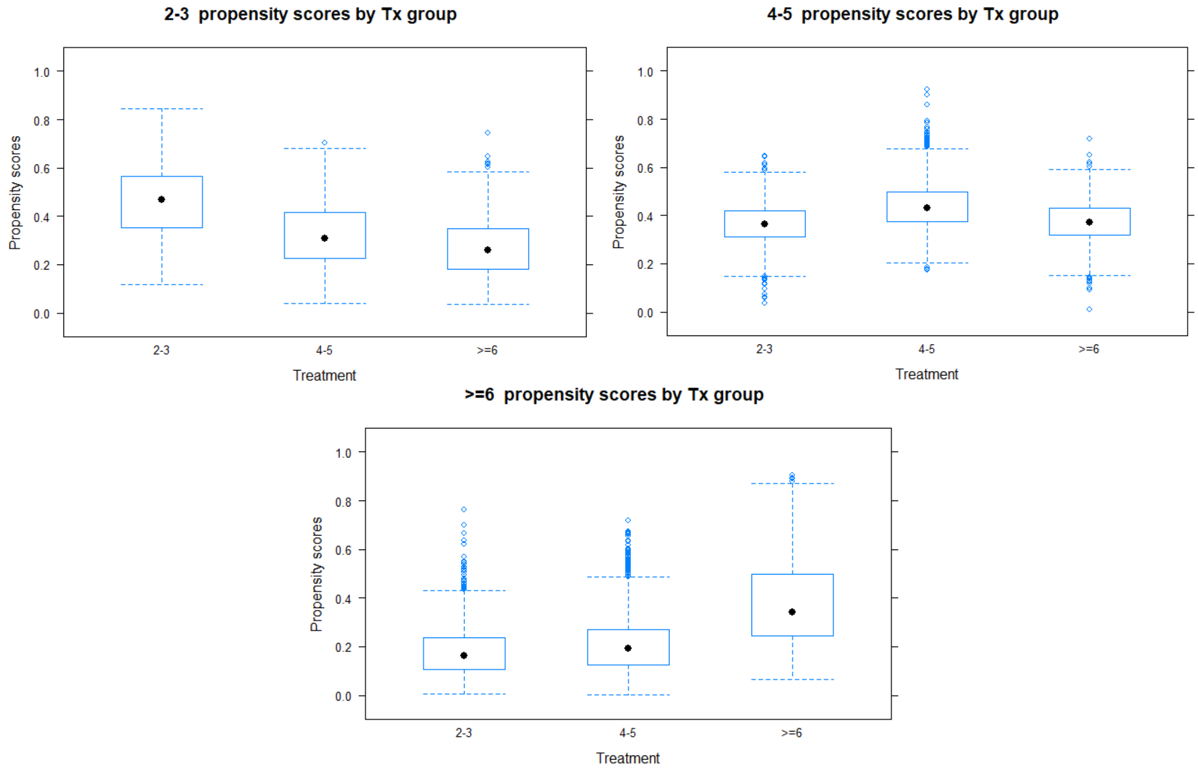

Supplementary Text 1: Propensity score matching analysis methodology

Propensity score weighting analysis was performed using the “mnps” function in the “Twang” package [1] in RStudio. Full details of the codes used could be found in the following tutorials [2, 3]. In summary, five steps were taken in performing the analysis.

1. Selection of a-priori variables or confounders

Based on the literature review [4, 5] and clinical knowledge of the delivery of neonatal care in the UK, a set of variables or confounders that are known to be associated with bronchopulmonary dysplasia or death in premature infants, which occurred before the allocation of the three postnatal dexamethasone (PND) treatment groups were determined a-priori. These a-priori variables are described in **Supplementary Table 2**.

2. Estimation of propensity score

The generalised boosted model approach in the “mnps” function was used to estimate the propensity of the infants being assigned to each of the three PND treatment groups based on the a-priori variables (**Supplementary Table 2**). Generalised boosted model is a machine learning approach whereby multiple tree-based regression models are built in stages in an iterative manner [2]. This iterative approach allows it to capture complex and non-linear relationships between the treatment group assignment and a-priori variables without overfitting. It was found to be superior to standard logistic regression in estimating propensity scores for treatment allocation [2, 3]. Missing values (**Supplementary Table 3**) were controlled for by including missing value indicators and balancing rates of missingness in the three groups (step 4).

Four stopping rules for the iterative process of the gradient boosted model approach were explored: mean absolute standardised mean difference (ASMD), max ASMD, mean Kolmogorov-Smirnov (KS)

and max KS. The mean ASMD stopping rule was used as it provided the best balance among the three groups with the second largest effective sample size.

3. Inverse probability treatment weighted (IPTW)

The propensity scores estimated by the gradient boosted model approach were then used as inverse weights in estimating the treatment effect, whereby the weight of the infant is determined by the inverse of the probability of the infant being allocated to the treatment group. The average treatment effect estimand [6] was used for the IPTW approach as the study aimed at assessing the change in the outcome if all infants in the study had been assigned to a particular PND treatment group relative to another group [3, 6]. The use of IPTW prevents infants to be excluded from the analysis and was found to reduce bias more than other propensity score analysis approaches such as stratification and co-variate adjustment [7].

4. Balance assessment of the a-priori variables

Balance of the a-priori variables (**Supplementary Table 2**) across the three PND treatment groups after weighing was assessed by examining the overlap of the propensity score distribution across the three groups as well as using standardised mean differences (SMD) and variance ratios (VR). SMD <0.1 and VR 0.5–2.0 were used to indicate good balance [8]. The balance diagnostics were visualised using balance plots developed using the “cobalt” package [9]. Boxplots by treatment group were used to assess the overlap of propensity scores.

All the a-priori variables were found to be balanced across the three groups after weighting in our study with a median (range) SMD of 0.009 (0.0007–0.05) and VR of 1.04 (1.01–1.17) (**Supplementary Table 4** and **Supplementary Figure 1**). There was an overlap in the propensity score distribution across the three groups (**Supplementary Figure 2**).

5. Estimation of treatment effect

The difference in weighted means was used to estimate the association between the treatment group and the outcomes of interest as the a-priori variables were balanced across the three groups after IPTW.

References

1. Cefalu M, Ridgeway G, McCaffrey D, Morral A, Griffin BA, Burgette L. twang: Toolkit for Weighting and Analysis of Nonequivalent Groups. R package version 2.5. https://CRAN.R-project.org/package=twang. 2021.
2. Burgette L, Griffin BA, McCaffrey D. Propensity scores for multiple treatments: A tutorial for the mnps function in the twang package RAND Corporation., 2021.
3. McCaffrey DF, Griffin BA, Almirall D, Slaughter ME, Ramchand R, Burgette LF. A tutorial on propensity score estimation for multiple treatments using generalized boosted models. *Stat Med* 2013: 32(19): 3388-3414.
4. National Institute for Health and Care Excellence. Specialist neonatal respiratory care for babies born preterm (NG124). NICE 2019.
5. British Association of Perinatal Medicine. Perinatal Management of Extreme Preterm Birth before 27 weeks of gestation. A Framework for Practice. October 2019. https://www.bapm.org/resources/80-perinatal-management-of-extreme-preterm-birth-before-27-weeks-of-gestation-2019 . [Date last accessed: October 12 2020].
6. Benedetto U, Head SJ, Angelini GD, Blackstone EH. Statistical primer: propensity score matching and its alternatives. *Eur J Cardiothorac Surg* 2018: 53(6): 1112-1117.
7. Austin PC. The performance of different propensity score methods for estimating marginal hazard ratios. *Stat Med* 2013: 32(16): 2837-2849.
8. Austin PC. An Introduction to Propensity Score Methods for Reducing the Effects of Confounding in Observational Studies. *Multivariate Behav Res* 2011: 46(3): 399-424.
9. Greifer N. cobalt: Covariate Balance Tables and Plots. R package version 4.3.2. https://CRAN.R-project.org/package=cobalt. 2022.
