# Supplementary material for: Impact of postnatal dexamethasone timing on preterm mortality and bronchopulmonary dysplasia: a propensity score analysis
Source: Eur Respir J. 2023 Oct 19;62(4):2300825. doi: 10.1183/13993003.00825-2023 (PMC10586235; doi:10.1183/13993003.00825-2023)
Supplement: Supplementary file 2 [file ERJ-00825-2023.Shareable.pdf]

EUROPEAN RESPIRATORY JOURNAL
ORIGINAL RESEARCH ARTICLE
T.C. KWOK ET AL.

SHAREABLE PDF

Impact of postnatal dexamethasone timing on preterm mortality and bronchopulmonary dysplasia: a propensity score analysis

T'ng Chang Kwok, Lisa Szatkowski and Don Sharkey

Centre for Perinatal Research, Population and Lifespan Science, School of Medicine, University of Nottingham, Queen's Medical Centre, Nottingham, UK.

Corresponding author: Don Sharkey (don.sharkey@nottingham.ac.uk)

Check for updates

Shareable abstract (@ERSpublications)
The ideal timing of postnatal dexamethasone to prevent BPD is unclear. After minimising residual confounding, first dexamethasone use at >5 weeks old is associated with a higher incidence of severe BPD/death and later extubation than use at 2–3 weeks old. https://bit.ly/3s6UtOT

Cite this article as: Kwok TC, Szatkowski L, Sharkey D. Impact of postnatal dexamethasone timing on preterm mortality and bronchopulmonary dysplasia: a propensity score analysis. *Eur Respir J* 2023; 62: 2300825 [DOI: 10.1183/13993003.00825-2023].

This extracted version can be shared freely online.

Copyright ©The authors 2023.

This version is distributed under the terms of the Creative Commons Attribution Licence 4.0.

This article has an editorial commentary: https://doi.org/10.1183/13993003.01473-2023

Received: 20 Feb 2023
Accepted: 8 Aug 2023

Abstract

Background Postnatal dexamethasone (PND) is used in high-risk preterm infants after the first week of life to facilitate extubation and prevent bronchopulmonary dysplasia (BPD) but the optimal treatment timing remains unclear. Our objective was to explore the association between the timing of PND commencement and mortality and respiratory outcomes.

Methods This was a retrospective National Neonatal Research Database study of 84 440 premature infants born <32 weeks gestational age from 2010 to 2020 in England and Wales. Propensity score weighting analysis was used to explore the impact of PND commenced at three time-points (2–3 weeks ($PND^{2/3}$), 4–5 weeks ($PND^{4/5}$) and after 5 weeks (PND^{6+}) chronological age) on the primary composite outcome of death before neonatal discharge and/or severe BPD (defined as respiratory pressure support at 36 weeks) alongside other secondary respiratory outcomes.

Results 3469 infants received PND. Compared with $PND^{2/3}$, infants receiving PND^{6+} were more likely to die and/or develop severe BPD (OR 1.68, 95% CI 1.28–2.21), extubate at later postmenstrual age (mean difference 3.1 weeks, 95% CI 2.9–3.4 weeks), potentially require respiratory support at discharge (OR 1.34, 95% CI 1.06–1.70) but had lower mortality before discharge (OR 0.38, 95% CI 0.29–0.51). $PND^{4/5}$ was not associated with severe BPD or discharge respiratory support.

Conclusions PND treatment after 5 weeks of age was associated with worse respiratory outcomes although residual bias cannot be excluded. A definitive clinical trial to determine the optimal PND treatment window, based on early objective measures to identify high-risk infants, is needed.
